# Isolation of infectious *Theileria parva* sporozoites secreted by infected *Rhipicephalus appendiculatus* ticks into an in vitro tick feeding system

**DOI:** 10.1186/s13071-021-05120-7

**Published:** 2021-12-24

**Authors:** Rubikah Vimonish, Kelcey D. Dinkel, Lindsay M. Fry, Wendell C. Johnson, Janaina Capelli-Peixoto, Reginaldo G. Bastos, Glen A. Scoles, Donald P. Knowles, Maxime Madder, George Chaka, Massaro W. Ueti

**Affiliations:** 1 Program in Vector-Borne Diseases, Department of Veterinary Microbiology and Pathology, Washington State University, Pullman, WA 99164-7040 USA; 2 USDA-ARS-Animal Diseases Research Unit, Pullman, WA 99164-6630 USA; 3 Paul G. Allen School for Global Animal Health, Washington State University, Pullman, WA 99164 USA; 4 Clinglobal, Black River, Tamarin, 90903 Mauritius; 5 Centre for Ticks and Tick-Borne Diseases, 309200 Lilongwe, Malawi; 6 Present Address: Invasive Insect Biocontrol and Behavior Lab, USDA, ARS, Beltsville, MD 20705 USA

**Keywords:** In vitro tick feeding system, *Theileria parva*, *Rhipicephalus appendiculatus*

## Abstract

**Background:**

Vector-borne diseases pose an increasing threat to global food security. Vaccines, diagnostic tests, and therapeutics are urgently needed for tick-borne diseases that affect livestock. However, the inability to obtain significant quantities of pathogen stages derived from ticks has hindered research. In vitro methods to isolate pathogens from infected tick vectors are paramount to advance transcriptomic, proteomic, and biochemical characterizations of tick-borne pathogens.

**Methods:**

Nymphs of *Rhipicephalus appendiculatus* were infected with *Theileria parva* by feeding on a calf during an acute infection. Isolation of sporozoites was accomplished by feeding infected adult ticks on an in vitro tick feeding system. Sporozoite viability was tested using in vitro bovine lymphocytes.

**Results:**

We isolated infectious *T. parva* sporozoites secreted into an in vitro tick feeding system. Infected adult *R. appendiculatus* ticks attached to and successfully fed on silicone membranes in the in vitro tick feeding system. Bovine blood in the receptacle was replaced with cell-free medium and the ticks were allowed to feed for 3 h to collect secreted *T. parva* sporozoites. Secreted sporozoites infected in vitro bovine lymphocytes, demonstrating that isolated sporozoites remained viable and infectious.

**Conclusions:**

This work is the first to report the isolation of mature infectious *T. parva* sporozoites using an in vitro tick feeding system, which represents a significant step towards the development of a more efficient control strategy for *T. parva*. Isolation of infectious tick-stage parasites will facilitate the examination of the vector-pathogen interface, thereby accelerating the development of next-generation vaccines and treatment interventions for tick-borne pathogens.

**Graphical Abstract:**

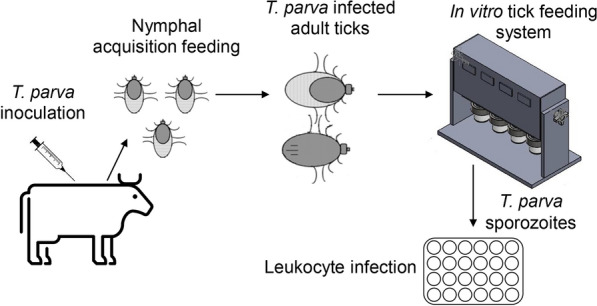

**Supplementary Information:**

The online version contains supplementary material available at 10.1186/s13071-021-05120-7.

## Background

Vector-borne diseases pose an increasing threat to animal health due to the movement of live animals and increasing geographic distribution of competent ticks [1–4]. Ticks are obligate, hematophagous ectoparasitic arachnids capable of transmitting a variety of pathogens to mammalian hosts, including viruses, bacteria, and protozoan parasites [5–7]. Pathogen acquisition occurs when ticks feed on an infected animal. After ingestion of the blood meal, pathogens enter midgut epithelial cells and undergo development, with subsequent migration to, and invasion of, the salivary glands where the pathogen replicates [8]. Following molting and transfer to a subsequent host, tick feeding stimulates pathogens to undergo a developmental cycle in tick salivary gland acini cells. Replication and development of pathogens in the midgut and salivary glands illustrates that ticks are efficient biological vectors [8–10].

The complex relationship between protozoan parasites and tick vectors is exemplified by *Theileria parva*, the etiologic agent of East Coast fever (ECF), and *Rhipicephalus appendiculatus* ticks, the parasite’s biological vector [9]. Following acquisition of *T. parva* by *R. appendiculatus* nymphal or larval ticks, sexual stage reproduction and maturation occurs in the midgut as ticks molt to the next instar. During this time, *T. parva* proceeds through multiple life stages [11]. Eventually, kinetes form and invade various organs, including salivary gland acini cells. Within acini cells, kinetes transform into immature sporozoites [12]. When the infected tick attaches to and feeds on a new host, sporozoites mature into the infectious form, and are transmitted to cattle or buffalo via saliva [12, 13]. Sporozoites invade lymphocytes and induce proliferation, resulting in ECF and high mortality of infected cattle [14]. The lack of methods to evaluate the interface between tick feeding and *T. parva* inoculation of the mammalian host has limited the study of pathogen transmission mechanisms. Previous studies on *T. parva* sporozoites used material isolated from tick salivary glands. However, the population of sporozoites isolated by dissection may contain earlier developmental stages that are not completely mature, and isolating mature secreted *T. parva* sporozoites might lead to clearer insights regarding the pathogenesis of this important infectious stage. Unfortunately, there are no available in vitro systems to isolate fully mature tick-specific stages of *T. parva* parasites.

To address this problem, we developed a novel method to obtain mature infectious sporozoites in cell-free medium by employing an in vitro tick feeding system. We demonstrated that isolated *T. parva* sporozoites from the in vitro tick feeding system were viable and infectious to bovine peripheral blood mononuclear cells (PBMC). To our knowledge, this is the first study that isolates mature, infectious *T. parva* sporozoites using an in vitro tick feeding system.

## Methods

### *Theileria parva*-infected ticks

The *R. appendiculatus* colony at the Animal Disease Research Unit of the United States Department of Agriculture’s Agricultural Research Service (USDA-ARS) was started from 50 male and 50 female ticks received in 2013 from Dr. Ivan Morrison at the Roslin Institute in Edinburgh, Scotland. At that time the colony had been maintained at the Roslin Institute for 25 years; ticks for the colony at Roslin were originally collected from Muguga [15]. A calf was subcutaneously inoculated with a stabilate of *R. appendiculatus* salivary glands infected with *T. parva* Muguga sporozoites, as described previously [16, 17]. The animal was monitored for clinical signs of disease and by polymerase chain reaction (PCR) to confirm infection with *T. parva*. Genomic DNA was extracted to determine infection using Tp104-PCR. Forward (5′-CGC CTG AGC CAA AAG CTA GTA-3′) and reverse (5′-TTC GAT GGC CTC GGT GAT T-3′) primers were designed to amplify a fragment of 149 base pairs. Reactions were performed in 25 μl containing 2 μl of template DNA, 0.4 µM of each primer, and 12.5 µl of RedTaq (Sigma-Aldrich, St. Louis, MO, USA) under the following conditions: one cycle at 95 °C for 3 min, 35 cycles of 95 °C for 30 s, 56 °C for 20 s and 72 °C for 30 s, with a final extension at 72 °C for 5 min. Amplicons were analyzed by 2% agarose gel electrophoresis. Amplicons were sequenced to verify parasite specificity (Eurofins Genomics, Louisville, KY, USA).

When the animal became febrile, developed mild peripheral lymphadenopathy, and was PCR-positive for *T. parva* in peripheral blood, approximately 1000 *R. appendiculatus* nymphs were applied under a cloth patch on the back of a calf and allowed to feed to repletion. Replete nymphs were collected, washed in tap water, and incubated at 26 °C and 93% relative humidity (RH) to molt to adults. Molted adult ticks were maintained at 15 °C and 93% RH and used in the in vitro tick feeding system within 2 to 4 months.

This study was approved by the Institutional Animal Care and Use Committee of the University of Idaho (Moscow, ID, USA), in accordance with the recommendations of the US Animal Welfare Act (United States Code, Title 7, Chapter 54, sections 2131–2159) and Animal Welfare Regulations (Code of Federal Regulations, Title 9, Chapter 1, Subchapter A, parts 1–4). The *T. parva-*infected animal developed severe ECF and was euthanized by intravenous injection of sodium pentobarbital (Fatal-Plus, Vortech Pharmaceuticals, USA).

### Detecting ticks infected with *T. parva*

Infected adult ticks were fed in the in vitro tick feeding system to allow *T. parva* sporozoite development in salivary gland acini cells. Adult ticks were fed blood at a constant 37 °C temperature. After 5 days of feeding, 23 ticks were dissected, salivary glands harvested, and genomic DNA extracted. The salivary gland infection rate and number of parasites were determined by PCR, as described above, and Tp104 quantitative PCR (Tp104-qPCR). Briefly, a primer set, forward (5′CAG ATG GAA GTG AAG TGT 3′) and reverse (5′ TAA ATG AAC AAG TGA TGC 3′), was designed to amplify a 101-base-pair fragment. A standard curve was constructed by amplification of 10^6^, 10^5^, 10^4^, 10^3^, and 10^2^ plasmid copies of the Tp104 gene as previously described [18]. The amplification reaction was performed in three technical replicates in a final volume of 20 µl using 0.4 µM of each primer, 1.5 µl of a template, and 10 µl of SsoFast™ EvaGreen^®^ Supermix. The qPCR conditions consisted of an initial cycle at 95 °C for 3 min, followed by 40 cycles at 95 °C for 30 s, 60 °C for 30 s, and 72 °C for 30 s. The number of *T. parva* sporozoites is presented as the average log of the triplicate values. CFX Manager™ software (Bio-Rad) was used to acquire the qPCR data. Standard deviations were calculated using Microsoft Excel.

### Pathogen localization within salivary glands

The presence of *T. parva* in acini cells was examined using immunohistochemistry (IHC) as previously described [19]. After sporozoite stimulation in the in vitro tick feeding system, infected ticks were fixed with 10% formalin and embedded in paraffin, and sequential 4 µm sections were deparaffinized in Clear-Rite and hydrated using an ethanol (100–70%) gradient. Sections were treated with citrate solution, pH 6 (Zymed, Carlsbad, CA, USA) for antigen retrieval and steamed for 30 min. The sections were stained using 2 µg/ml of anti-PIM (IgG2a) or anti-P67 (IgG1) monoclonal antibodies [20]. Monoclonal antibodies anti-ANA8 (IgG1) [21] and anti-F16 (IgG2a) [22] were used as negative isotype controls. To detect primary antibody binding, sections were incubated with horseradish peroxidase-labeled anti-mouse immunoglobulin (Dako Corp., Carpinteria, CA, USA) and 3-amino-9-ethylcarbazole containing hydrogen peroxide. Sections were counterstained with Mayer’s hematoxylin. Sections were examined using a Nikon Eclipse microscope.

### Isolation of in vitro secreted *T. parva* sporozoites

Fifty *R. appendiculatus* adults (25 males and 25 females) infected with *T. parva* were applied to the silicone membrane of the in vitro tick feeding system as described previously [23]. The ticks were allowed to feed initially on uninfected bovine blood at a packed cell volume of 10% containing 1× antibiotic/antimycotic solution (Sigma-Aldrich, St. Louis, MO, USA). Adult ticks were fed blood at a constant 37 °C temperature. After 21 h, the blood receptacle was washed three times with distilled water, once with 70% ethyl alcohol, and twice with phosphate-buffered saline (PBS) to remove blood residue. Four milliliters of cell-free complete RPMI medium at 37 °C containing 10% fetal bovine serum, 24 mM of HEPES, 2 mM of l-glutamine, and 10 µg/ml of gentamicin was added to the blood receptacle. Ticks were fed for 3 h. Subsequently, the medium was collected, and sporozoites recovered by centrifugation at 5000×*g* for 2 min. Blood was replaced to the blood receptacle, and ticks were fed for 21 h. The process was repeated for six consecutive days. Genomic DNA was extracted to determine the number of secreted sporozoites by Tp104-qPCR as described above.

### Infectivity of secreted *T. parva* sporozoites

Infectivity of secreted *T. parva* sporozoites was determined by incubating isolated parasites with bovine PBMC from an uninfected animal in vitro [24, 25]. Sixty ml of whole bovine blood from an uninfected animal was collected into EDTA and centrifuged at 1200×*g* for 10 min at room temperature. The buffy coat was recovered and suspended in Hank's Balanced Salt Solution. The cells were overlaid onto Histopaque 1077 (Sigma-Aldrich, St. Louis, MO, USA) and centrifuged at 900×*g* for 30 min. Cells were collected from the Histopaque interface, washed three times, suspended in 5 ml of complete RPMI 1640 (Gibco, Gaithersburg, MD, USA) supplemented with 10% calf bovine serum, 20 mM HEPES buffer (Gibco), 50 µM β-mercaptoethanol (Gibco), 2 mM L-glutamine (Gibco), and 50 µg/ml gentamicin (Gibco). PBMC were infected as previously described [24] by exposing 10^6^ bovine cells per well with ~ 10^5^ secreted *T. parva* sporozoites and incubating at 37 °C with 5% CO_2_. Secreted *T. parva* sporozoites were collected on day 5 post-tick attachment.

*Theileria parva*-exposed lymphocytes were analyzed at different time points after in vitro infection using flow cytometry and immunocytochemistry (ICC). For flow cytometry, harvested cells were washed with PBS, fixed, and permeabilized with fixation/permeabilization solution (BD Cytofix/Cytoperm™ Plus Fixation/Permeabilization Kit) (BD BioSciences) for 20 min at 4 °C. The fixed cells were washed twice with BD Perm/Wash buffer (BD Cytofix/Cytoperm™ Plus Fixation/Permeabilization Kit) (BD BioSciences). Cells were stained on ice with 2.5 μg/ml anti-*T. parva* PIM monoclonal antibody [20] conjugated with DyLight 650 for 30 min in the dark. Autologous uninfected lymphocytes exposed to 5 µg/ml Concanavalin A (ConA) (Sigma-Aldrich) and stained with anti-PIM monoclonal antibody were used as a negative control. Stained cells were washed twice with Perm/Wash buffer and acquired with a Guava easyCyte flow cytometer using InCyte guavaSoft 3.1.1 (Luminex). The data were analyzed with FCS Express version 6 (De Novo Software, Pasadena, CA, USA) to provide the percentage of total cells by quadrant.

For ICC, exposed cells were collected and cytospun at 1000 rpm for 3 min onto positively charged glass slides (Fisher Scientific, Waltham, MA, USA). Fixed cells were probed with 1 μg/ml of anti-PIM or isotype control anti-F16 monoclonal antibody [22]. Horseradish peroxidase-labeled anti-mouse immunoglobulin and 3-amino-9-ethylcarbazole containing hydrogen peroxide was used to detect immunoglobulin binding. Lymphocytes were counterstained with Mayer's hematoxylin (Sigma-Aldrich). Slides were examined using a Nikon Eclipse microscope.

### Titration of secreted *T.parva* sporozoite infectivity

Tenfold serial dilutions of secreted *T. parva* sporozoites were performed, and sporozoites combined immediately with 10^6^ bovine PBMC as described above [24, 25]. PBMC were incubated at 37 °C with 5% CO_2_ and *T. parva*-infected lymphocytes analyzed by flow cytometry at different time points after in vitro infection and ICC as described above.

## Results

### Infection of *R. appendiculatus* ticks with *T. parva*

**Fig. 1:**
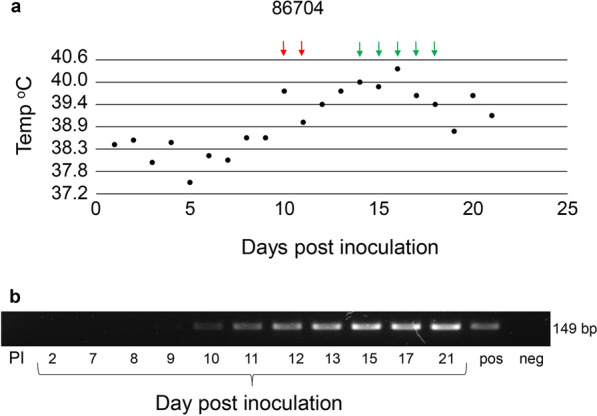
Infection of *R. appendiculatus* via acquisition feeding on a *T. parva*-infected calf. **a** Calf was infected via subcutaneous inoculation with *T. parva* salivary gland stabilate. The calf developed severe fever beginning 10 days post-infection. Red arrow indicates nymphal tick application and green arrows indicate collection of replete nymphs. **b** Detection of *T. parva* via p104 PCR. Amplicons were visualized in 2% agarose gel

*Rhipicephalus appendiculatus* nymphal ticks were acquisition-fed on a calf exhibiting clinical signs of ECF including fever, enlarged lymph nodes, and anorexia. Nymphal ticks were applied on day 10, the first day of fever and Tp104-PCR positivity (Fig. 1a), and on day 11. The animal remained Tp104-PCR positive during nymphal tick feeding (Fig. 1b). Replete nymphs were collected from the infected animal and incubated at 26 °C and 93% RH to molt to the adult stage.

### Detecting ticks infected with *T. parva*

**Fig. 2:**
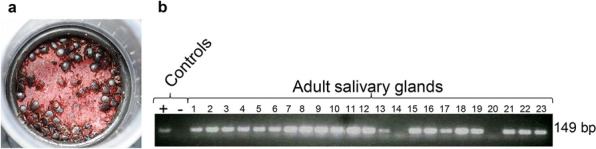
Infected *R. appendiculatus* adult ticks feeding on a silicone membrane. **a** A representation of adult ticks attached to a silicone membrane. **b** Detection of tick salivary gland infected with *T. parva* via p104 PCR. Amplicons were visualized in 2% agarose gel

**Fig. 3:**
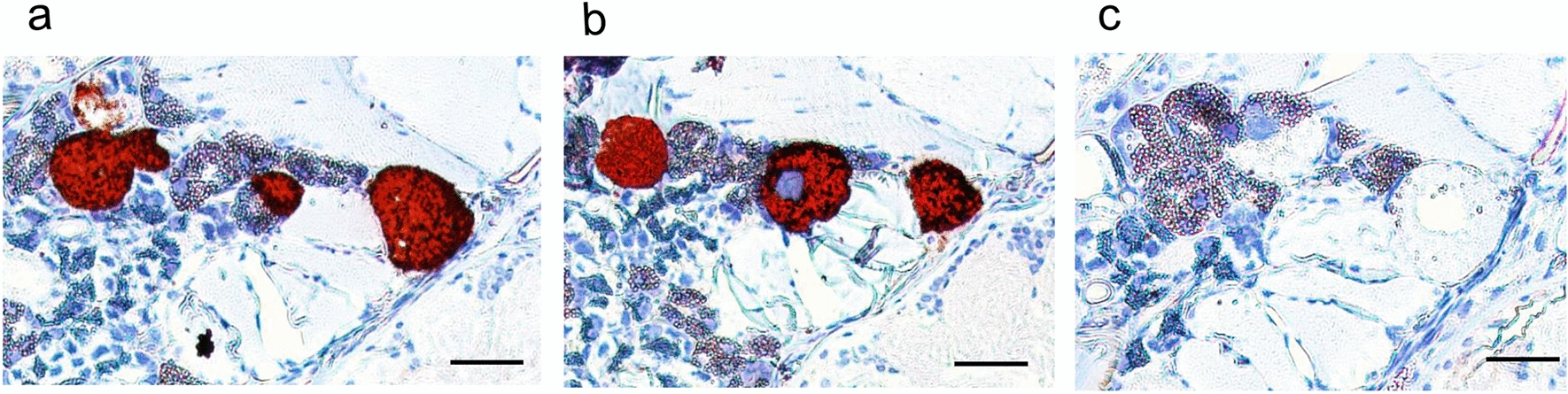
Immunohistochemical detection of *T. parva* salivary gland acinus colonization in adult ticks. Tick sections were probed with monoclonal antibodies: **a** anti-PIM; **b** anti-p67; **c** isotype control. Red indicates antibody-specific reactivity to *T. parva* colonies. Scale bar: 50 µm

Freshly molted adult ticks were applied to the in vitro tick feeding system, and approximately 90% attached to the silicone membrane within 24 h (Fig. 2a). A cohort of adult ticks fed for 5 days showed that salivary glands from 21 of 23 ticks were Tp104-PCR positive (Fig. 2b), an infection rate greater than 91%. Tick salivary gland pairs contained an average of 10^4.23 (±1.8)^ parasites. *Theileria parva* colonization of acini cells was demonstrated by the reactivity of anti-p67 and anti-PIM monoclonal antibodies in thin sections using IHC (Fig. 3a and b). Isotype controls showed no reactivity to salivary glands infected with *T. parva* (Fig. 3c).

### Isolation of secreted *T. parva* sporozoites

In the first 24 h of in vitro tick feeding, no secreted sporozoites were detected by Tp104-qPCR. In trial 1, we detected secretion of *T. parva* at 48 h. In trials 2 and 3, secreted parasites were detected at day 4 post-tick attachment (Table 1). The level of *T. parva* sporozoites secreted in 3 h and collected daily varied between 10^3^ and 10^6^/ml (Table 1).

**Table 1:** *Theileria parva* sporozoites secreted into cell-free medium in three independent trials

Tick feeding (days)	*T. parva *sporozoites per milliliter of cell-free medium		
	Trial 1	Trial 2	Trial 3
1	nd	nd	nd
2	7.2 × 10^4^	nd	nd
3	6.5 × 10^5^	nd	nd
4	4.9 × 10^4^	1.2 × 10^6^	3.1 × 10^5^
5	3.3 × 10^3^	2.6 × 10^6^	6.5 × 10^5^
6	nd	4.7 × 10^6^	1.1 × 10^6^

nd: no Tp104-qPCR detection

### Infectivity of secreted *T. parva* sporozoites

**Fig. 4:**
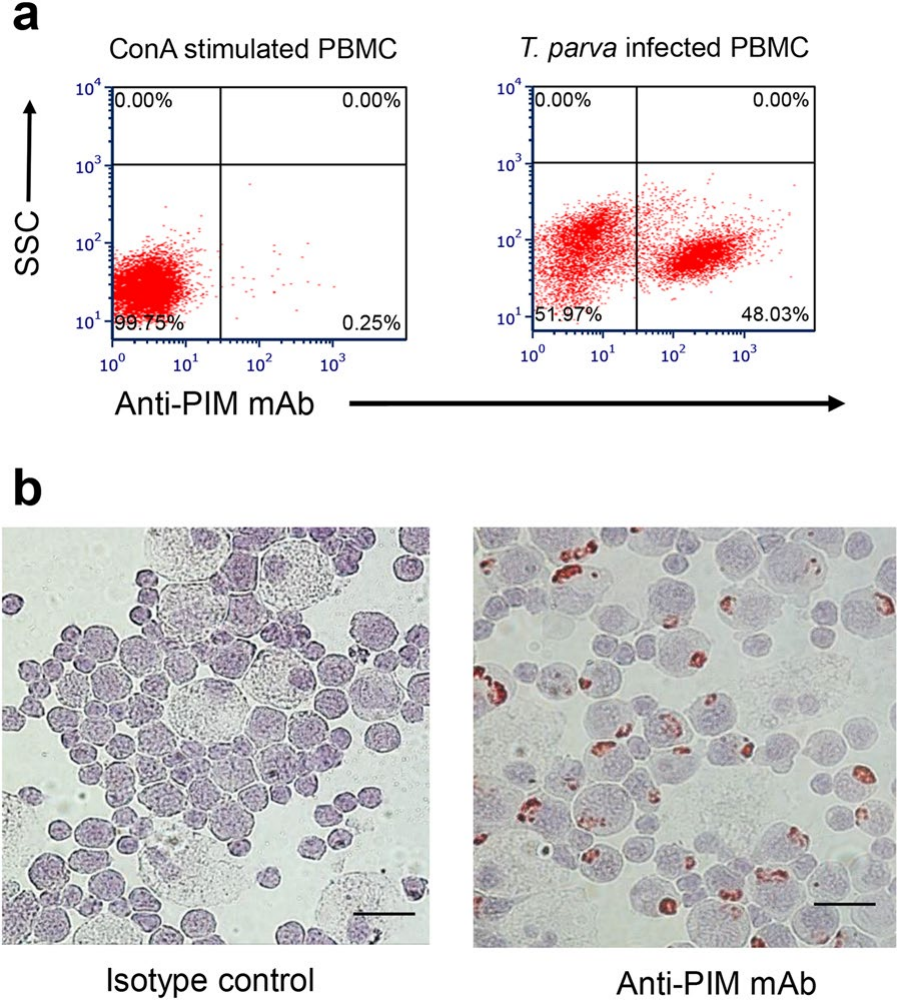
Demonstration of infectivity of secreted *T. parva* sporozoites collected from the in vitro tick feeding system. **a** Flow cytometric detection of cultured *T. parva*-infected lymphocytes. **b** Immunocytochemistry demonstrating *T. parva* schizont formation in bovine lymphocytes. Lymphocytes were probed with monoclonal antibodies: left panel, isotype control; right panel, anti-PIM. Red indicates antibody-specific reactivity to *T. parva* within bovine lymphocytes. Scale bar: 20 µm

Infectivity of secreted sporozoites was determined in vitro by incubation with bovine PBMC. Dense groups of cells were observed after 1 week of incubation (data not shown). In the second week, *T. parva* infection was determined by flow cytometric analysis. Approximately 48% of the cells were infected with *T. parva* (Fig. 4a). The ICC assay confirmed the formation of schizonts in lymphocytes (Fig. 4b).

### Titration of secreted *T.* parva sporozoites

**Fig. 5:**
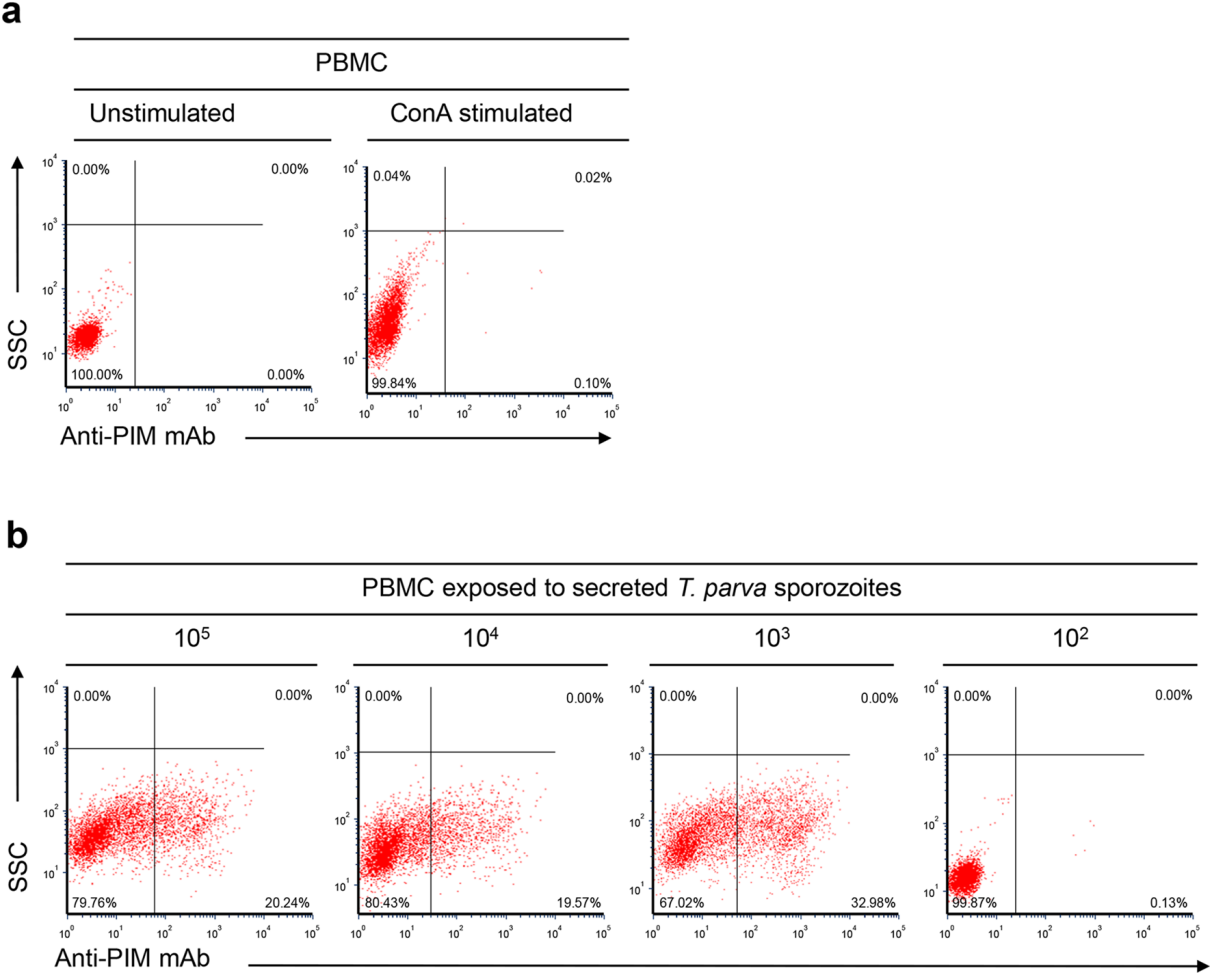
Determination of the minimum dose of *T. parva* sporozoites from the in vitro tick feeding system sufficient to infect bovine lymphocytes in vitro. Lymphocytes were probed with anti-PIM monoclonal antibody. **a** Unstimulated and ConA-stimulated negative controls. **b** Flow cytometric detection of infected lymphocytes exposed to tenfold serial dilutions of *T. parva* sporozoites isolated from the in vitro tick feeding system

Infection of in vitro lymphocytes by 10^3^, 10^4^, and 10^5^ secreted sporozoites was 32.98%, 19.57%, and 20.24%, respectively (Fig. 5 bottom panel). However, 10^2^ secreted sporozoites failed to infect lymphocytes. The proliferation of *T. parva*-infected lymphocytes continued for at least 5 weeks of incubation. During this time, the number of uninfected control lymphocytes decreased dramatically. The formation of *T. parva* schizonts in lymphocytes was demonstrated by anti-PIM antibody reactivity. Anti-p67 and isotype control antibodies did not react with infected lymphocytes (Additional file 1: Figure S1).

## Discussion

In this study, we tested two hypotheses. The first was that *T. parva*-infected *R. appendiculatus* ticks secrete sporozoites into cell-free medium during feeding in an in vitro tick feeding system. The second was that secreted *T. parva* sporozoites remain infectious to lymphocytes. The data described herein, whereby *T. parva-*infected *R. appendiculatus* adult ticks fed on silicone membranes, allowed the collection of secreted *T. parva* sporozoites, and that the secreted sporozoites subsequently infected bovine lymphocytes in vitro, supports both hypotheses. To the authors’ knowledge, this is the first study to isolate secreted and infectious *T. parva* sporozoites utilizing an in vitro tick feeding system. This system will facilitate transcriptomic, proteomic, and biochemical characterizations of parasite tick-specific stages that may lead to the development of novel intervention strategies for tick-borne diseases.

*Theileria parva* infects vertebrate and invertebrate hosts to complete its life cycle. After acquisition by *R. appendiculatus* nymphal ticks, parasite maturation occurs in the midgut lumen where the parasite proceeds through multiple life stages and eventually spreads to various tick organs, including tick salivary gland acini cells [26, 27]. During tick feeding, sporozoites mature to the infectious form [12], are inoculated via tick saliva, resulting in severe ECF [26]. The development of an in vitro system to isolate *T. parva* sporozoites will advance the improvement of control strategies for ECF.

In this study, all trials using uninfected and *T. parva*-infected *R. appendiculatus* adult ticks consistently showed an average rate of ~ 90% tick attachment to silicone membranes and the ticks fed continuously on the membrane. Similar results were obtained in a previous study performed by Waladde et al. using *R. appendiculatus* nymphs that fed to repletion [28]. Other studies using artificial membrane feeding systems have demonstrated tick attachment rates varying between 32 and 100% [29, 30]. In the current study, continuous in vitro tick feeding on the silicone membrane permitted the isolation of secreted *T. parva* sporozoites, additional evidence of tick feeding success.

Previous studies showed that feeding infected ticks stimulated *T. parva* colonization of salivary gland E-cells of type III acini [31]. Additionally, an in vivo study showed sporogony completion and development of mature sporozoites after a few days of feeding on an animal [13]. An interesting finding in our study was that sporozoites were secreted into the cell-free medium within 2 days of attachment to the silicone membrane and were continuously secreted for up to 4 days. Our results are consistent with pathogen replication in salivary gland acini cells, culminating in transmission that is dependent upon resumption of tick feeding on a subsequent animal. Similar to *T. parva* development in tick salivary glands stimulated by feeding infected ticks on rabbits, feeding infected ticks on the in vitro tick feeding system also stimulated sporozoite development in tick salivary glands. However, a potential caveat of the in vitro tick feeding system is the scalability in producing enough biological material for generating vaccines. The capacity of the in vitro tick feeding system is up to 600 adult ticks per unit (Btissam et al., 2021, Assessment of the in vitro tick feeding system for the successful feeding of adult *Rhipicephalus appendiculatus* ticks, submitted manuscript), which is greater than the number of ticks feeding on a single rabbit that generally receive 400 adult ticks for *T. parva* sporozoites stimulation. The use of this in vitro tick feeding system may reduce the number of animals used for ECF vaccine production in the infection and treatment (ITM) method [32].

To demonstrate the infectivity of secreted *T. parva* sporozoites for bovine PBMC, we performed serial dilutions of secreted sporozoites per previous in vitro studies that used parasites from ground infected ticks [33, 34]. To reduce potential immature parasite forms, *T. parva* sporozoites were collected from the in vitro tick feeding system on day 5 post-tick attachment. We detected schizont formation in *T. parva* sporozoite-exposed bovine lymphocytes by ICC using anti-PIM antibodies. Previous studies demonstrated that both PIM and p67 are important for sporozoite invasion [16, 35]. Our results confirmed that after sporozoite invasion, p67 was not essential for parasite development inside lymphocytes, as it was no longer detected (Additional file 1: Figure S1). A previous study detected low p67 expression in the *T. parva* schizont stage [36]. After sporozoite invasion, PIM remained expressed by schizonts, and it has been postulated to be involved in the establishment of schizonts in lymphocytes [4]. Our results are consistent with previous descriptions that, upon *T. parva* schizont development, changes such as uncontrolled cell proliferation in bovine leukocyte behavior occur. In our study, infected lymphocytes replicated for several months, indicating that, after the establishment of in vitro infection, the cells became immortalized. To improve the number of mature sporozoites secreted into the cell-free medium, a heat stimulation step of infected ticks may be necessary before feeding in the in vitro tick feeding system. Previous studies demonstrated that heat-stimulated infected *R. appendiculatus* resulted in *T. parva* maturing in salivary glands [37, 38].

## Conclusions

This work is the first to report the isolation of mature infectious *T. parva* sporozoites using an in vitro tick feeding system. It represents a significant step towards the development of a more efficient control strategy for ECF. The use of the in vitro tick feeding system to produce the sporozoite vaccine may reduce the need for feeding of ticks on rabbits to stimulate parasite development within tick salivary gland acini cells before dissection and harvesting of sporozoites. The method presented herein not only provides a system that can be used for isolation of mature sporozoites for use in tick-borne pathogen vaccine development, but also offers a framework for the replacement, reduction, and refinement of animals in the production of the ITM live vaccination method, representing a potentially significant advance in the welfare of animals used to study human and animal tick and tick-borne pathogens. Additionally, it should be noted that this system could allow the collection and identification of tick salivary gland-secreted immunomodulating proteins, furthering the understanding of the tick-mammalian host interface.

## Supplementary Information

**Additional file 1: Figure S1.** Immunocytochemistry demonstrating *T. parva* schizont expressing PIM but not P67. Infected lymphocytes were probed with monoclonal antibodies: **a** Isotype control, **b** Anti-PIM, and **c** Anti-P67. Red indicates antibody-specific reactivity to *T. parva* within bovine lymphocytes. Scale bar: 20 µm.

## Data Availability

The data supporting the conclusions of this article are included within the article and its additional files.

## Abbreviations

ECF: East Coast Fever
PIM: Polymorphic immunodominant molecule
ICC: Immunocytochemistry
IHC: Immunohistochemistry
PCR: Polymerase chain reaction
qPCR: Quantitative polymerase chain reaction
PBMC: Peripheral blood mononuclear cell
DNA: Deoxyribonucleic acid
RH: Relative humidity
HEPES: 4-(2-Hydroxyethyl)-1-piperazineethanesulfonic acid
PBS: Phosphate-buffered saline
ConA: Concanavalin A
ITM: Infection and treatment
USDA-ARS: United States Department of Agriculture-Agricultural Research Service
