# Supplementary figures and images for: Isolation of infectious Theileria parva sporozoites secreted by infected Rhipicephalus appendiculatus ticks into an in vitro tick feeding system
Source: Parasit Vectors. 2021 Dec 24;14:616. doi: 10.1186/s13071-021-05120-7 (PMC8704063; doi:10.1186/s13071-021-05120-7)

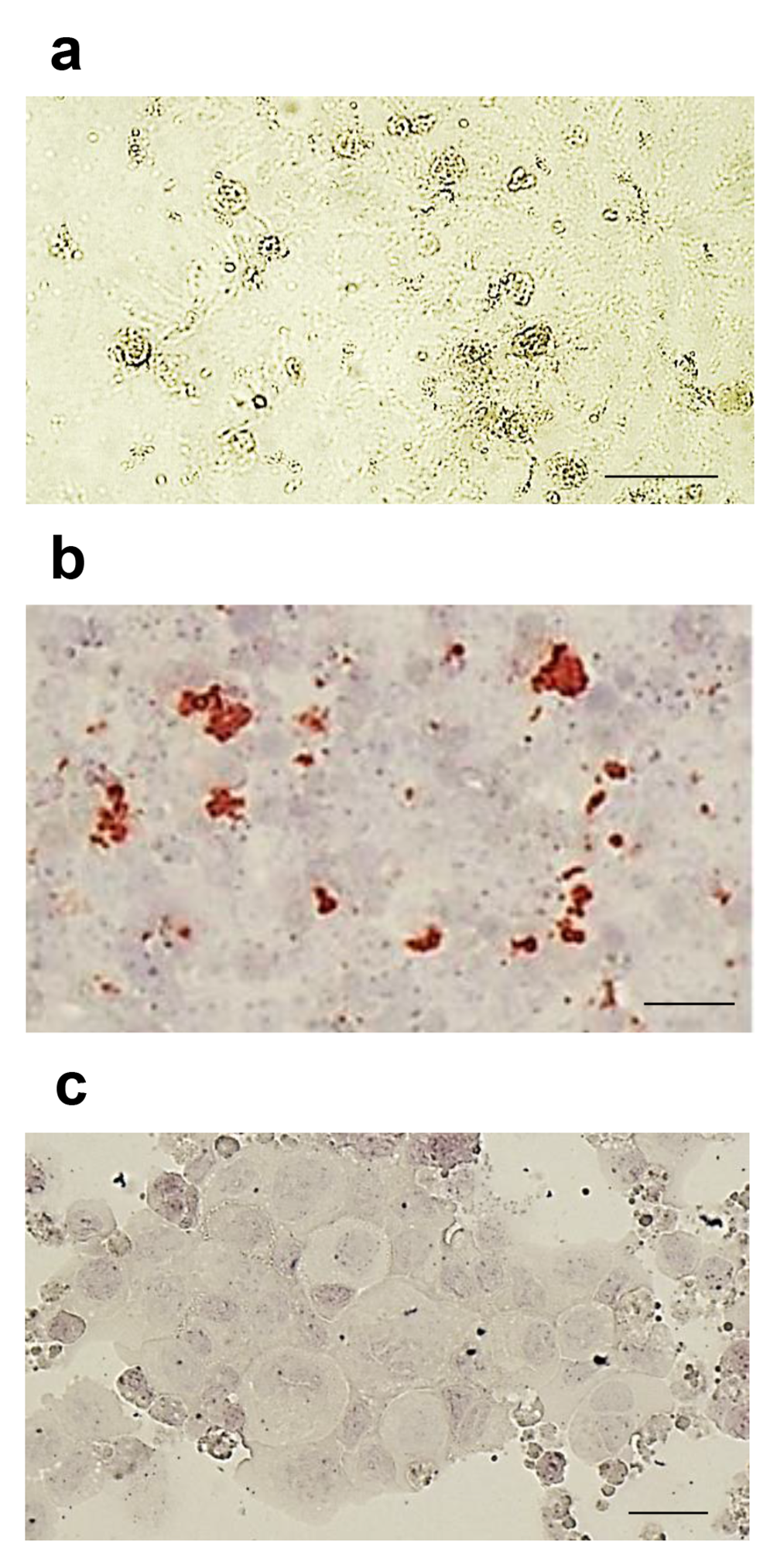

Supplement: Supplementary file 1 — Additional file 1: Figure S1. Immunocytochemistry demonstrating T. parva schizont expressing PIM but not P67. Infected lymphocytes were probed with monoclonal antibodies: a Isotype control, b Anti-PIM, and c Anti-P67. Red indicates antibody-specific reactivity to T. parva within bovine lymphocytes. Scale bar: 20 µm. [file 13071_2021_5120_MOESM1_ESM.tif]
